# Evaluation of sperm retrieval rate with bilateral testicular sperm extraction in infertile patients with azoospermia

**Published:** 2015-11

**Authors:** Mohammad Reza Moein, Mahmoud Reza Moein, Jalal Ghasemzadeh, Soheila Pourmasoumi

**Affiliations:** 1 *Research and Clinical Center for Infertility, Shahid Sadoughi University of Medical Sciences, Yazd, Iran.*; 2 *Faculty of Medicine, Shahid Sadoughi University of Medical Sciences, Yazd, Iran.*

**Keywords:** *Infertility*, *Azoospermia*, *Testicular sperm extraction*

## Abstract

**Background:**

About 10% to 15% of infertile men have azoospermia, which could be obstructive or non-obstructive. Diagnostic biopsy from the testis and recently testicular sperm extraction (TESE) are the most precise investigations in these patients. Testicular biopsy can be done unilaterally or bilaterally. The worth of unilateral or bilateral testicular biopsy in men with azoospermia is controversial.

**Objective:**

To evaluate the necessity of bilateral diagnostic biopsy from the testis in new era of diagnosis and treatment of male infertility.

**Materials and Methods:**

In this retrospective study, we reviewed the results of testis biopsy in 419 azoospermic men, referred to Yazd Research and Clinical Center for Infertility from 2009-2013. Patients with known obstructive azoospermia were excluded from the study.

**Results:**

In totally, 254 infertile men (60.6%) were underwent unilateral TESE, which in 175 patients (88.4%) sperm were extracted from their testes successfully. Bilateral testis biopsy was done in 165 patients (39.4%) which in 37 patients (22.4%), sperm were found in their testes tissues.

**Conclusion:**

Due to the low probability of positive bilateral TESE results especially when we can’t found sperm in the first side, we recommend that physicians re-evaluate the risk and benefit of this procedure in era of newer and more precise technique of sperm retrieval like micro TESE.

## Introduction

Infertility is the inability to conceive offspring within one year of unprotected intercourse (1). Infertility can be due to either male or female factors or both of them may be involved. The complete absence of spermatozoa in two semen analysis is defined as azoospermia (1). Azoospermia is found in 1% of all men (2, 3) and 10- 15% of infertile men (3). 

Respecting etiology, there are two types of azoospermia. One is obstructive azoospermia that is due to obstruction in male reproductive tract. Another type, which is also more common, is non-obstructive azoospermia that is due to inadequate production of sperm by testis. 

Physical examination, detailed medical history, hormonal analysis (FSH, testosterone) and genetic studies will help to determine the etiology of azoospermia and in most cases they can also determine the type of azoospermia (4). Obstructive azoospermia can be caused by: infections, trauma, surgery, radiation or congenital anomalies (5). Non-obstructive azoospermia can be induced by: drugs, genetic problems, congenital anomalies like varicocele and undescendent testis, radiation, and other less common factors such as heat, genital injuries which may affect sperm production (5).

In most instances, in men with non- obstructive azoospermia we need to do testicular sperm extraction (TESE) followed by intracytoplasmic sperm injection (ICSI).

TESE which is mainly used for sperm retrieval in patients with non-obstructive azoospermia can be done by different methods. One method is conventional TESE which usually done as an outpatients surgery without using any magnification and usually with local anesthesia. Another and more efficient method is micro TESE in which testis tissues are extracted selectively by guide of surgical microscope.

Open testicular biopsy appears to be more efficient than needle biopsy of testis Testicular Sperm Aspiration for the recovery of testicular spermatozoa in patients with non-obstructive azoospermia (6, 7). To obtain more sperm from the testicles in patients with non-obstructive azoospermia, sometimes it is necessary to do multiple TESE (8). Multiple TESE has shown to be associated with inflammatory changes and stable devascularization of testis (9).

Historically, it is advised that diagnostic biopsy from the testis is performed bilaterally. But necessity of unilateral versus bilateral diagnostic testicular biopsies in men with azoospermia is controversial. In a study by Plaset *et al*, they showed that bilateral testicular biopsies are better than unilateral biopsies in the assessment of men with azoospermia (10). Schulze *et al* in 1999 showed that there is a 32.7% difference in sperm retrieval rate between two testicles (11). With respect to reported complications of TESE and surgical operation like hematoma and decrease testis function, in this study we reviewed the results of bilateral TESE in azoospermic men referred to Yazd infertility center from 2009-2013 to evaluate the risks and benefits of this procedure.

## Materials and methods

In this retrospective cross sectional study, we gathered the data of all infertile men with azoospermia who were referred to Yazd Research and Clinical Center for Infertility from 2009-2013 and were candidates for testis biopsy. Ethic committee of Yazd Research and Clinical Center for Infertility approved this study. Because we just reviewed the data retrospectively and had no contact with the patients at the time of our study, so no any consent were taken from patients.

Medical history was taken and genital physical exam was done for all of patients. Men with known obstructive azoospermia like patients with history of vasectomy or men with absence of vas deferens in physical examination and those who had been candidate for percutaneous epididymal sperm extraction were excluded from the study. Patients who had only one palpable testis also were excluded from this study.

Formal testis biopsy without any magnification was done by two physicians with similar method with no internal quality control. Local anesthesia achieved by injecting spermatic cord and scrotal skin with 10 ml of 2% lidocaine solution. Through 1 cm scrotal incision, scrotal layers and tunica albuginea were incised and a small testis tissue was removed and sent to andrology lab to search for sperm. If the first sample was negative for sperm, at least two other samples were taken from other parts of the same testis. Biopsy was taken from the other side if all these samples were negative. In andrology lab, testicular tissues were put directly into a Petri dish containing Hamm's F-10 medium, then testicular tissues were crushed and ragged using sterile glass slides and then examined at once under an inverted microscope, using× 400 magnification for the presence and motility of testicular spermatozoa.

Then, the entire petri dish was searched, when no spermatozoa were observed, testicular tissues with medium were collected into 5 ml Falcon tubes and centrifuged for 5 min at 300 g. This procedure was repeated two times. The pellet was re-suspended in 50 μl of Hamm's F-10 medium, and then second look for spermatozoa was performed.

Tunical and scrotal layers were sutured with absorbable 4-0 chromic suture. Sperm freezing was done for patients in whom sperm was found and they were scheduled for later ICSI.

**Statistical analysis**

No statistical analysis was done because we had only single set of data and no comparison was made.

## Results

**Figure 1:**
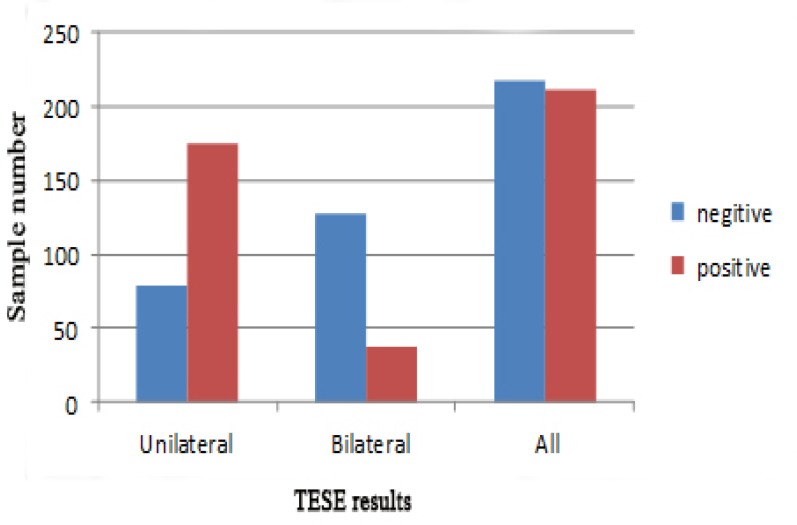
Comparison of Testicular sperm extraction results in unilateral and bilateral samples

**Figure 2:**
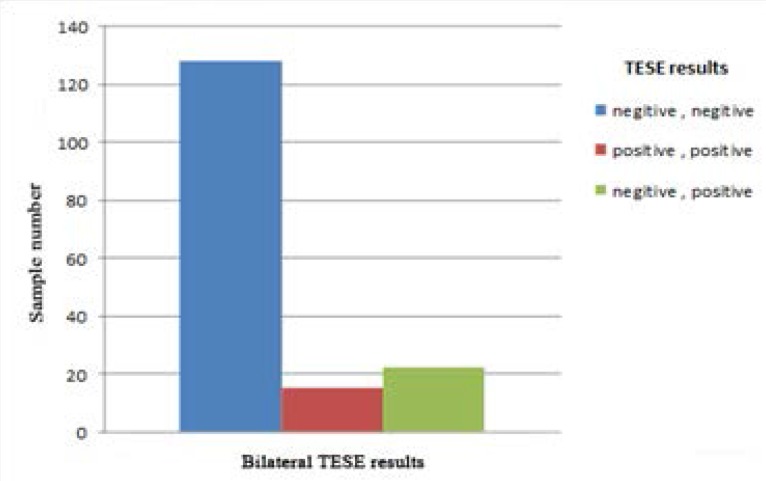
Comparison of TESE results in bilateral samples.

Totally, testis biopsy results of 419 non-obstructive azoospermic men were studied. Mean age of patients was 31.65 ± 6.78 years (range: 20 to 64 years). Overall, positive results were seen in 212 samples (50.6%). In 175 patients (88.4%) (of 254 patients (60.6%) who underwent unilateral TESE), sperm could be extracted from their testicles successfully. Bilateral testis biopsy was done in 165 patients (39.4%) but only in 37 men (22.4%), sperm was found in their testicles and 128 (77.6%) patients had no sperm at all. In 15 patients (9.1%), samples from both testicles were positive. In 22 patients (13.3%) no sperm was found in one testis, but TESE samples from other testis were positive (Figure 1, 2).

## Discussion

There are two types of azoospermia. Obstructive azoospermia is usually due to reproductive tract obstruction and constitute about one third of cases of azoospermia. The other type is non-obstructive azoospermia in which there is inadequate sperm production in testis to be seen in ejaculate (12). Physical examination, detailed medical history and hormonal analysis (FSH, testosterone) although may help us to determine the type of azoospermia, but in most instances we can’t determine the type of azoospermia accurately and need more precise diagnostic methods. For many years open testis biopsy was the most reliable and accurate method for differentiating obstructive from non- obstructive azoospermia (13). After introduction of ICSI in 1991, different methods for sperm retrieval were popularized. Methods like percutaneous epididymal sperm aspiration (PESA) and microscopic epididymal sperm aspiration (MESA) are used for sperm retrieval in obstructive azoospermia and testicular sperm aspiration and TESE have used extensively in non-obstructive azoospermia for sperm retrieval (14). Ability to freeze sperm cells which are retrieved by these above methods give the patients a chance to get rid of psychological stress related to the second operation before microinjection. So, diagnostic testis biopsy in most instances was replaced by new techniques of sperm retrieval. The use of TESE in men with obstruction for ICSI was first introduced in 1993 (8, 9). A few years later, this method (TESE-ICSI), was used successfully for patients with non-obstructive azoospermia (9, 15). In this method which could be done as an outpatient procedure with local anesthesia, a small incision is made over testis and samples of testis tissues are extracted and sent for andrology lab (16). Open testicular biopsy appears to be more efficient than needle biopsy for the recovery of testicular spermatozoa in azoospermic patient with non-obstructive azoospermia (9, 13) also fertilization and embryo transfer rates were higher, when ICSI is done with testicular sperm in these patients (17). To obtain more sperm from the testicles in patients with non-obstructive azoospermia, sometimes it is necessary to do multiple TESE (14). Multiple TESE of course may also be associated with inflammatory changes and stable devascularization (15). But the value of unilateral or bilateral testicular biopsies in patients with azoospermia is still controversial. In a study conducted by Plas *et al*, they stated that bilateral testicular biopsies are superior to unilateral biopsies in azoospermia patients, and they recommended bilateral testicular biopsies for them (10). In another study Ramasamy and colleague’s showed that only 40 of the 506 men (8%) who underwent bilateral testicular microdissection had sperm found on the contralateral side when no sperm were identified on the initial side (18). Our study showed that in more than 75% of patients who were underwent bilateral TESE no sperm could be found. These findings indicate that the same pathological process underlying the defective spermatogenesis in both testicles. Moreover in only 13.3% patients we were able to find sperm in another side although no sperm were found in the first side. We also know that TESE and testis biopsy may have some complications like irreversible testis injury, pain, hematoma and infection (16). Also some studies have shown spermatogenesis will stop for 6 to 8 weeks after TESE along with adhesion in testis (9). Indeed, it has shown that repetition of testicular surgeries decreases the chance of finding sperm in subsequent testicular sperm retrieval procedures (17). So, with respect to very low probability of sperm retrieval in such cases and possibility of newer and more efficient techniques of sperm retrieval like micro-TESE, we recommend that for diagnostic purpose and in formal TESE procedures only one testis be evaluated and if the first side was negative for sperm leave the other side and refer the patients to infertility centers with more sophisticated and advanced facilities.

## Conclusion

It seems that, we should be more careful about the inherent superiority of the bilateral TESE, in comparison with unilateral biopsies in azoospermic patients in era of more new and efficient ways for sperm retrieval and preservation and new techniques of ART.

## note

***This article extracted from M.D. thesis. (Mahmoud Reza Moein)***
